# Cardiac STAT3 Deficiency Impairs Contractility and Metabolic Homeostasis in Hypertension

**DOI:** 10.3389/fphar.2016.00436

**Published:** 2016-11-16

**Authors:** Raffaele Altara, Romain Harmancey, Sean P. Didion, George W. Booz, Fouad A. Zouein

**Affiliations:** ^1^Department of Pharmacology and Toxicology, School of Medicine, University of Mississippi Medical Center, JacksonMS, USA; ^2^Department of Physiology and Biophysics, School of Medicine, University of Mississippi Medical Center, JacksonMS, USA; ^3^Department of Pharmacology and Toxicology, Faculty of Medicine, American University of BeirutBeirut, Lebanon

**Keywords:** cardiac hypertrophy, hypertension, mitochondria, cardiac function, fatty acid oxidation, metabolism

## Abstract

Signal transducer and activator of transcription 3 (STAT3) protects the heart from acute ischemic stress. However, the importance of STAT3 to the heart in chronic stress, such as hypertension, is not known. To study this, we used cardiomyocyte-targeted STAT3 knockout (KO) mice and Angiotensin II (ANG II) infusion by osmotic minipumps. After 4 weeks, ANG II induced similar cardiac hypertrophy in wild type (WT) and cardiac Cre-expressing control (CTRL) mice with no impairment of cardiac function. In contrast, STAT3 KO mice exhibited reduced contractile function but similar hypertrophy to CTRL mice. Ejection fraction and fractional shortening decreased by 22.5 and 27.3%, respectively. Since STAT3 has direct protective effects on mitochondrial function, we examined rates of glucose and oleate oxidation by isolated perfused hearts using a Langendorff system. Hearts of ANG II-treated STAT3 KO and CTRL mice had similar rates of oleate oxidation as saline-infused WT mice. Rates of glucose oxidation were similar between hearts of WT plus saline and CTRL plus ANG II mice; however, glucose oxidation was increased by 66% in hearts of ANG II-treated STAT3 KO mice. The ratio of maximal ATP yield from glucose to fatty acid oxidation was 21.1 ± 3.1 in hearts of ANG II-treated STAT3 KO mice vs. 12.6 ± 2.2 in hearts of ANG II-treated CTRL mice. Lactate production was also elevated in hearts of ANG II-treated STAT3 KO mice by 162% compared to ANG II-treated CTRL mice. Our findings indicate that STAT3 is important for maintaining contractile function and metabolic homeostasis in the hypertensive heart, and STAT3 deficiency promotes a switch toward glucose utilization.

## Introduction

Heart Failure is a leading cause of death in the USA and developed world, and while survival after diagnosis has improved over time, the death rate remains high with ∼50% mortality within 5 years of diagnosis (Roger et al., 2012). The total estimated annual cost of heart failure is >30 billion dollars and that figure is expected to more than double over the next decade due to an aging population (Heidenreich et al., 2013). Hypertension is the second most common risk factor for heart failure and accounts for ∼25% of heart failure cases (Kannel and Cobb, 1992). In the elderly, as many as 68% of heart failure cases are linked to hypertension and community-based studies indicate hypertension contributes to heart failure in 60% of patients (Yamasaki et al., 2003).

Accumulating evidence indicates that preserving bioenergetics, including maintaining mitochondrial fatty acid oxidation (FAO), is critical for cardiac function and preventing both ischemic and non-ischemic heart failure (Abdurrachim et al., 2015; Kundu et al., 2015). Hypertension-induced cardiac hypertrophy is associated with a reduction in FAO and increased reliance on anaerobic glycolysis (Kolwicz et al., 2012). The basis for the metabolic reprogramming of the hypertrophied and early stage failing heart is not understood, but seems to involve both transcriptional and mitochondrial post-transcriptional events (Lai et al., 2014).

Signal transducer and activator of transcription 3 (STAT3) was originally identified as a transcription factor and was subsequently shown to be important in the heart for inducing a protective gene program associated with delayed preconditioning of myocardium (Kurdi and Booz, 2007; Zouein et al., 2015). The importance of STAT3 in protecting the heart from chronic stresses, such as hypertension, is not known. Additionally, STAT3 is now known to have protective actions that do not involve gene expression. Two sites of phosphorylation in the C-terminal transcription activation domain (TAD) of STAT3 constitute an on-switch for its activation (Zouein et al., 2015). Y705 phosphorylation in canonical signaling leads to gene expression. S727 phosphorylation enhances gene expression, but also has novel genomic and non-genomic actions that are independent of Y705 phosphorylation. We recently reported that mice with a global S727A mutation in STAT3 that precludes phosphorylation, devoid of a baseline phenotype, exhibit a loss of cardiac myocytes and diminished contractile function in response to the chronic stress of pressure overload due to hypertension (Zouein et al., 2013). Our findings suggest that STAT3 protects the heart from the harmful effects of hypertension, but the basis for this protection is not known.

The non-genomic actions of STAT3 include unexplained direct protective actions on the mitochondrial electron transport chain (ETC). STAT3 is present in heart mitochondria and reduced STAT3 content impairs respiratory function, reduces activity of complex I, and increases susceptibility of the mitochondrial permeability transition pore (mPTP) to open, a harbinger of cell death (Zouein et al., 2015). In this study, we assessed the importance of cardiac myocyte-specific STAT3 in maintaining the homeostasis of cardiac energy metabolism during hypertension using mice expressing a STAT3 in the heart lacking the domain important for both its mitochondrial and genomic actions.

## Materials and Methods

### Materials

Angiotensin II (ANG II) acetate was from Bachem (Torrance, CA, USA). Probumin bovine serum albumin fatty acid-free was from EMD Millipore (Billerica, MA, USA). PerkinElmer was the source for [9,3-^3^H]oleate and [U-^14^C]glucose. Insulin (Humulin R U-100) was from Eli Lilly and Company (Indianapolis, IN, USA). The lactate assay kit (MAK064) was from Sigma-Aldrich (St. Louis, MO, USA).

### Animals

**FIGURE 1:**
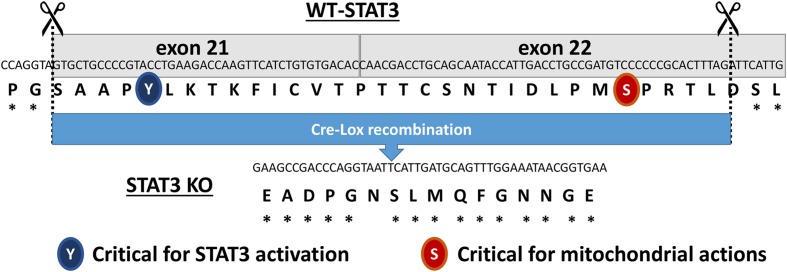
**Diagram showing the excised region of the *Signal Transducer and Activator of Transcription 3* (*STAT3*) gene in the cardiac myocyte-targeted STAT3 knockout (KO) mice.** The two regulatory sites of phosphorylation (Y705 and S727) are highlighted.

The STAT3 floxed mice were originally produced by Takeda et al. (1998). Briefly, STAT3 KO mice that express a functionally deficient STAT3 lacking the C-terminus TAD selectively in cardiac myocytes were generated by crossing *STAT3* floxed (exons 21 and 22) mice with mice expressing Cre recombinase under the control of the αMHC promoter (**Figure 1**). The STAT3 KO mice were homozygous for *loxP*-flanked STAT3 alleles and hemizygous for the *Cre* transgene. Further validation of the generated STAT3 KO mice is reported in the Supplementary Material (**Supplementary Figure 1**). Age-matched (2–4 months old) wild type mice and cardiomyocyte-expressing Cre recombinase mice were used as controls (CTRLs). Mice were on the C57BL/6 background (αMyHC-Cre; The Jackson Laboratory, Bar Harbor, ME, USA) and studies were performed on male mice. Animals were maintained on 8640 Teklad 22/5 rodent diet (Harlan Laboratories, Indianapolis, IN, USA).

### Study Protocol

The study complied with the Guide for the Care and Use of Laboratory Animals: Eighth Edition (National Academy of Sciences Institute for Laboratory Animal Research, 2011) and was approved by the Institutional Animal Care and Use Committee of UMMC (protocol #1192B). Saline (0.9%) or ANG II (1000 ng/kg/min) was delivered via Alzet miniosmotic pumps (Model 1004) implanted subcutaneously for 28 days. Animals were treated with ibuprofen (4 mg/Kg IP) as analgesic for 1 day following implantation of pumps.

### Echocardiography

Cardiac function was assessed by echocardiography using a VEVO 770 high resolution *in vivo* imaging system from VisualSonics. Echocardiography was performed prior to pump implantation, at the midway point, and at study completion. Measurements were made with a 707B RMV scanhead with a center frequency of 25 MHz and frequency band ranging from 12.5 to 37.5 MHz. Mice were anesthetized with 1.5% isoflurane and placed on pre-warmed EKG transducer pad with heart rate, body temperature, and EKG monitored. Two-dimensional B-mode parasternal long axis views were obtained first to visualize aortic and mitral valves. The transducer was then rotated clockwise 90° to obtain the parasternal short axis view. Ejection fraction (EF) and fractional shortening (FS) were determined from the M-mode images.

### Fatty Acid and Glucose (Glc) Oxidation

*Ex vivo* cardiac metabolic analyses were performed as previously described (Wu et al., 2015). Briefly, mice were anesthetized with Inactin hydrate (150 mg/kg IP) and anticoagulated with heparin (40 USP IP). Hearts were rapidly excised and arrested in ice-cold Krebs–Henseleit buffer. Hearts were mounted on a Langendorff apparatus and retrogradely perfused at a constant coronary perfusion pressure of 70 mmHg with non-recirculating Krebs–Henseleit buffer containing Glc (5.5 mmol/L), sodium oleate (0.4 mmol/L) bound to bovine serum albumin, [9,3-^3^H] oleate (0.2 μCi/mL), [U-^14^C] Glc (0.1 μCi/mL), and insulin (40 μU/mL). The buffer was equilibrated with 95% O_2_–5% CO_2_ and maintained at 37°C. After stabilization for 30 min, rates of oleate and Glc oxidation were determined by quantitative collection of [^3^H]_2_O and [^14^C]O_2_ from the coronary effluent.

### Statistics

Values are reported as mean ± SEM for n number of independent observations. For single comparisons, statistical significance was determined by a Student’s *t*-test. For multiple comparisons, one-way or two-way ANOVA followed by an appropriate *post hoc* test was used. Statistical significance was taken as *p* ≤ 0.05.

## Results

### Cardiac STAT3 Deletion Impairs Contractility with Hypertension, but Does Not Prevent Hypertrophy

**FIGURE 2:**
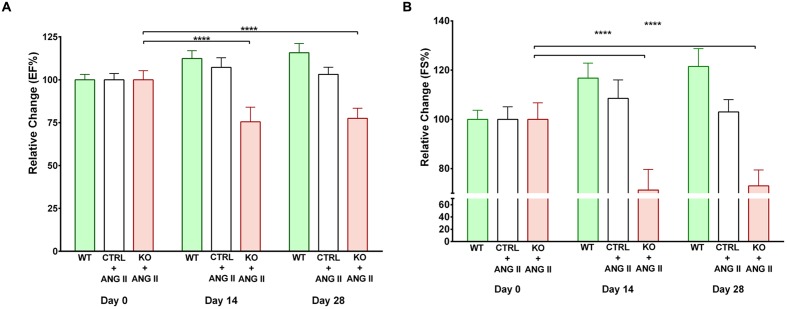
**Effect of STAT3 deletion on cardiac function in Angiotensin II (ANG II-treated mice).** **(A)** Ejection fraction (EF) and **(B)** fractional shortening (FS) were assessed by echocardiography in control (CTRL) and STAT3 KO mice infused with ANG II and wild type (WT) mice infused with saline. Measurements were performed on day 0, 14, and 28 and presented as the relative change from day 0. ^∗∗∗∗^*p* ≤ 0.0001, *n* ≥ 11.

Angiotensin II (ANG II)-infusion of mice for 28 days was used as a model of hypertension-induced cardiac hypertrophy, which commonly precedes heart failure (Booz, 2007). Cardiac function was assessed by echocardiography. Contractile function was maintained by hearts of both WT and Cre^+^ mice treated with ANG II as assessed by EF and FS (**Supplementary Figure 2**). Since no difference was observed between WT and Cre^+^ mice for subsequent studies the two groups were pooled as CTRLs. The time course for the effect of ANG II on cardiac function is shown in **Figure 2**. Hearts of STAT3 KO mice exhibited a decline in contractile function that was already in evidence by day 14 (**Figure 2**). EF was reduced by 25.7 ± 7.0 % (*n* = 11, *p* = 0.001) and FS by 30.1 ± 8.0 % (*n* = 11, *p* = 0.001) at day 28 when compared to CTRLs. **Table 1** provides the EF and FS values at 28 days for all groups and shows that loss of cardiac STAT3 resulted in reduced EF and FS in response to ANG II infusion.

**Table 1:** Summary of cardiac function at study completion.

Cardiac Function	Group	Mean ± SEM	*P*
**LVEF (%)**	WT + Saline	57.33 ± 2.63	*ns*
	WT + ANG II	52.22 ± 3.27	
	CRE^+^ + Saline	57.26 ± 3.84	*ns*
	CRE^+^ + ANG II	54.34 ± 2.38	
	KO + Saline	58.84 ± 4.73	0.01
	KO + ANG II	39.40 ± 2.86	

**LVFS (%)**	WT + Saline	30.26 ± 1.81	*ns*
	WT + ANG II	27.11 ± 2.01	
	CRE^+^ + Saline	30.82 ± 3.23	*ns*
	CRE^+^ + ANG II	27.57 ± 1.50	
	KO + Saline	30.75 ± 3.37	0.0067
	KO + ANG II	21.22 ± 1.88	


*LVEF, Left ventricular ejection fraction; LVFS, Left ventricular fractional shortening; WT, Wild type mouse; CRE^+^, Cre recombinase positive mouse; KO, STAT3 knockout mouse; ANG II, Angiotensin II.*

**FIGURE 3:**
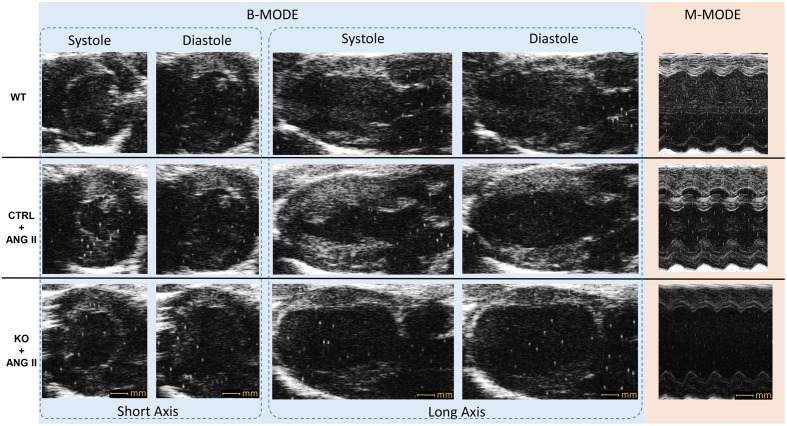
**Representative echocardiography B-mode and M-mode images.** CTRL and STAT3 KO mice were infused with ANG II for 28 days; WT mice were infused with saline. Compared to WT mice, CTRL and KO mice exhibited increased wall thickness.

**FIGURE 4:**
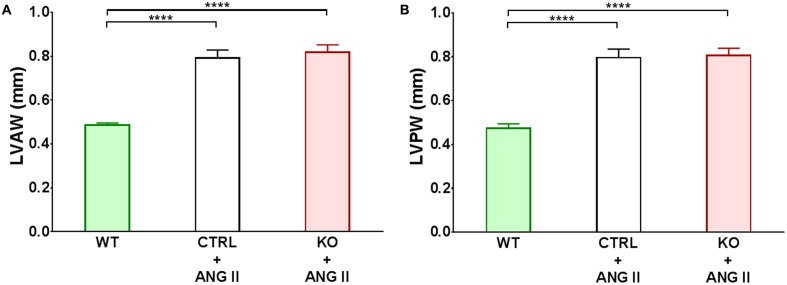
**Quantification of cardiac hypertrophy detected by echocardiography. Changes in left ventricular anterior wall (LVAW) and posterior wall (LVPW) at 28 days of treatment are shown in panels** **(A,B)**, respectively. ^∗∗∗∗^*p* ≤ 0.001, *n* ≥ 11.

**FIGURE 5:**
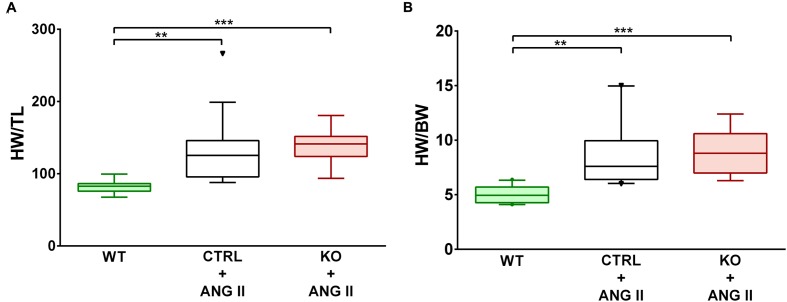
**ANG II-induced similar cardiac hypertrophy in CTRL and STAT3 KO mice. Mice were infused for 28 days with ANG II (CTRL and STAT3 KO) or saline (WT). Cardiac hypertrophy was assessed as** **(A)** heart weight to tibia length (HW/TL) ratio or **(B)** heart weight to body weight ratio (HW/BW). ^∗∗^*p* ≤ 0.01, ^∗∗∗^*p* ≤ 0.001, *n* ≥ 11.

Loss of cardiomyocyte STAT3 did not have an effect on the development of cardiac hypertrophy with ANG II. Cardiac anatomy evidenced by ultrasounds displayed a similar hypertrophic morphology for STAT3 KO and CTRL ANG II treated mice (**Figures 3** and **4**). ANG II infusion induced similar hypertrophy of STAT3 KO and CTRL hearts as assessed by an increase in the heart weight to tibia length (**Figure 5A**) and the heart weight to body weight ratio (**Figure 5B**).

### Cardiac STAT3 Deletion Does Not Impair FAO and Increases Glucose Oxidation

**FIGURE 6:**
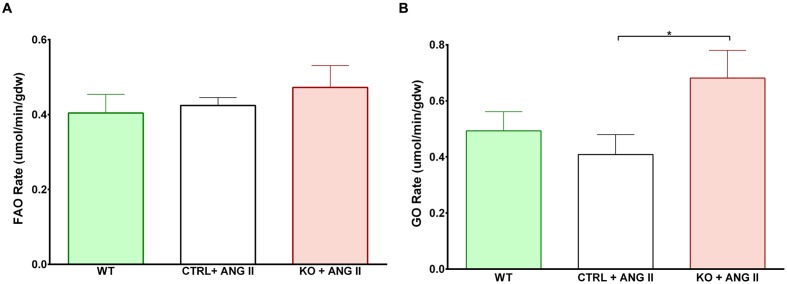
**Assessment of metabolism in perfused hearts. Mice were treated for 28 days with ANG II (CTRL or STAT3 KO) or saline (WT). Rates of** **(A)** fatty acid oxidation (FAO) and **(B)** glucose oxidation (GO) were assessed in hearts mounted on a Langendorff apparatus. ^∗^*p* ≤ 0.05, *n* ≥ 11. gdw, gram dry weight.

**FIGURE 7:**
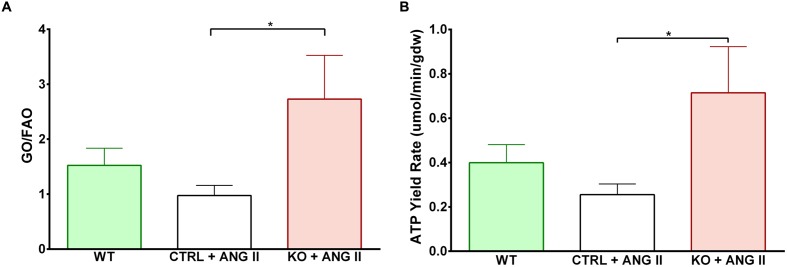
**Shift toward increased glucose oxidation over fatty acid oxidation in STAT3 KO hearts with ANG II treatment.** **(A)** Relative contribution of GO to FAO in hearts of CTRL and STAT3 KO mice infused with ANG II or WT mice infused with saline for 28 days. **(B)** Maximal ATP yield from glucose to fatty acid oxidation. ^∗^*p* ≤ 0.05, *n* ≥ 11.

**FIGURE 8:**
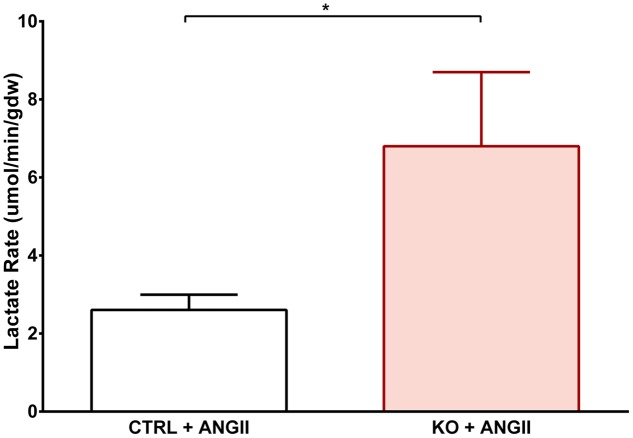
**Effect of STAT3 KO on lactate production in hypertrophied hearts.** CTRL and STAT3 KO mice were treated with ANG II for 28 days. Lactate production was assessed in hearts mounted on a Langendorff apparatus. ^∗^*p* ≤ 0.05, *n* ≥ 11.

Cardiac FAO was not affected by ANG II infusion in either CTRL or STAT3 KO mice (**Figure 6A**). In addition, no effect of ANG II on rates of Glc oxidation was seen in hearts of CTRL mice. However, as seen in **Figure 6B**, rates of Glc oxidation were enhanced in the hearts of STAT3 KO mice by 66% (*n* = 16, *p* = 0.018). Glc oxidation as a fraction of FAO was significantly increased by ANG II treatment in STAT3 KO hearts of mice compared to hearts of CTRL mice (**Figure 7A**) and the ratio of maximal ATP yield from Glc oxidation to FAO was increased with ANG II treatment by 68% in hearts of STAT3 KO mice compared to hearts of CTRL mice (**Figure 7B**). As seen in **Figure 8**, lactate production was also elevated in STAT3 KO hearts with ANG II infusion compared to CTRL hearts treated with ANG II.

## Discussion

Late stage cardiac hypertrophy and heart failure are associated with impaired mitochondrial FAO and a shift to greater reliance on Glc metabolism, which is thought to be inadequate for normal cardiac function (Lai et al., 2014). In our study, we observed that mitochondrial FAO is maintained for upward of 28 days with ANG II-induced hypertension and cardiac hypertrophy. Others previously reported that hearts of ANG II-infused mice do not show impaired FAO at 7 days (Mori et al., 2013). More significantly, FAO was not altered in the hearts of STAT3 KO mice treated with ANG II for 28 days, although these mice exhibited cardiac dysfunction at 14 days that persisted out to 28 days. Thus, we conclude that STAT3 is not important in the heart to maintain normal fatty acid utilization under chronic stress conditions of ANG II infusion, despite the fact that STAT3 deficiency has been reported to impair ETC function in the heart, including complex I and II activities (Wegrzyn et al., 2009).

We observed that Glc oxidation was increased in STAT3 KO mice treated with ANG II. Unexpectedly, FAO was not altered, which we would have predicted to decrease due to the Randle cycle (Heather and Clarke, 2011). Increased Glc oxidation in the face of unaltered FAO is further evidence that mitochondrial metabolic function is not adversely affected by STAT3 deletion in the heart under the chronic stress condition of cardiac hypertrophy. However, STAT3 loss may have compromised one or more regulatory mechanism associated with controlling the balance between Glc oxidation and FAO and precipitated the development of metabolic alterations associated with pathological remodeling of the heart. Our observation that hearts of STAT3 KO mice treated with ANG II produce more lactate than CTRL hearts is further evidence that glycolytic flux is enhanced in these hearts as well, as increased workload on the heart eventually leads to increased Glc uptake, glycogenolysis, glycolytic flux, and pyruvate oxidation (Depre et al., 1999). Under certain conditions, enhanced glycolysis has been associated with metabolic acidosis and impaired mechanical function of the heart (Clanachan, 2006; Hall et al., 2016), and therefore may have contributed to the impaired cardiac contractility that we observed in STAT3 KO mice treated with ANG II.

Our findings of contractile impairment and increased Glc oxidation in the absence of a decrease in FAO are reminiscent of results reported by Yan et al. (2009). In their study, increased Glc uptake and oxidation in the mouse heart overexpressing the Glc transporter GLUT1 was associated with cardiac dysfunction in diet-induced obesity in the absence of any change in the contribution of FAO to total substrate use. In addition, unlike WT hearts, these transgenic hearts failed to demonstrate a decrease in FAO when challenged by increased work load, suggesting a loss of metabolic flexibility. Moreover, hearts of transgenic mice fed a high fat diet were unable to sustain the high contractile performance associated with high workload. The increase reliance on Glc oxidation in the absence of any change in FAO was associated with oxidative stress and enhanced p38 activation, both of which have been implicated in contractile dysfunction.

Our study also reveals that cardiac myocyte expression of STAT3 is not important for the development of cardiac hypertrophy in response to ANG II-induced hypertension. No difference in the extent of cardiac hypertrophy was found between STAT3 KO and CTRL mice. Genetic deletion of IL-6 was recently reported to attenuate pressure overload-induced cardiac hypertrophy (Zhao et al., 2016). IL-6 is a strong inducer of STAT3 and by inference STAT3 was implicated in left ventricular hypertrophy and dysfunction in this study. However, IL-6 activates other signaling pathways that might contribute to cardiac hypertrophy, including several that affect calcium handling, a proven protagonist of cardiac myocyte hypertrophic growth. As discussed elsewhere (Kurdi and Booz, 2007), the conclusion the STAT3 couples to cardiac hypertrophy is largely based on circumstantial evidence obtained using cells in culture; definitive evidence that endogenous STAT3 is essential for physiological or pathological hypertrophy of the heart is lacking.

Our study has certain limitations. No difference was observed between WT and Cre^+^ mice in cardiac function with or without ANG II infusion. However, we cannot preclude the possibility that subtle differences were present at a subcellular level. Nonetheless, STAT3 KO hearts behaved differently from Cre^+^ expressing hearts with ANG II infusion, highlighting a unique role of this transcription factor in the heart under stress conditions. In addition, we performed our study with male mice since the degree of hypertension induced by ANG II is greater in male mice. Future studies will need to assess whether STAT3 has sex-specific effects in the heart. Plus, loss of STAT3 may have removed an ANG II-activated cardioprotective mechanism, rather than eliminated a broad based endogenous protective mechanism (Altara et al., 2016). Whether STAT3 protects the heart in other models of cardiac hypertrophy will need to be assessed. Finally, ANG II is known to have both blood pressure-dependent and -independent effects in the heart. Our study was not designed to discriminate between these two effects.

## Conclusion

Our results clearly show that mitochondrial function as assessed by both fatty acid and Glc oxidation is not adversely affected by STAT3 deletion following 4 weeks treatment with ANG II. Moreover, we report for the first time that cardiac myocyte-specific STAT3 is not important for hypertrophic growth in response to increased blood pressure. Therefore, our findings suggest that the direct non-genomic actions of STAT3 at the level of the mitochondria do not play a role in the development of heart failure in response to increased cardiac hypertrophy.

## Author Contributions

GB was involved in designing experiments, analyzing data and writing the manuscript. RH and SD were involved in designing critical experiments for the manuscript. FZ and RA supervised the study. All authors were involved in writing and reviewing the manuscript before submission.

## Conflict of Interest Statement

The authors declare that the research was conducted in the absence of any commercial or financial relationships that could be construed as a potential conflict of interest.

## References

[B1] Abdurrachim D. Luiken J. J. Nicolay K. Glatz J. F. Prompers J. J. Nabben M. (2015). Good and bad consequences of altered fatty acid metabolism in heart failure: evidence from mouse models. *Cardiovasc. Res.* 106 194–205. 10.1093/cvr/cvv105 25765936

[B2] Altara R. Didion S. P. Booz G. W. (2016). Conflicting mechanisms of AT2 cardioprotection revealed. *Cardiovasc. Res.* 112 426–428. 10.1093/cvr/cvw199 27659501 PMC5031951

[B3] Booz G. (2007). “Left ventricular physiology in hypertension,” in *Comprehensive Hypertension*, eds Lip Y. H. Hall J. E. (Maryland Heights, MO: Mosby).

[B4] Clanachan A. S. (2006). Contribution of protons to post-ischemic Na(+) and Ca(2+) overload and left ventricular mechanical dysfunction. *J. Cardiovasc. Electrophysiol.* 17(Suppl. 1), S141–S148. 10.1111/j.1540-8167.2006.00395.x 16686669

[B5] Depre C. Vanoverschelde J. L. Taegtmeyer H. (1999). Glucose for the heart. *Circulation* 99 578–588. 10.1161/01.CIR.99.4.578 9927407

[B6] Hall M. M. Rajasekaran S. Thomsen T. W. Peterson A. R. (2016). Lactate: friend or foe. *PM R* 8 S8–S15. 10.1016/j.pmrj.2015.10.018 26972271

[B7] Heather L. C. Clarke K. (2011). Metabolism, hypoxia and the diabetic heart. *J. Mol. Cell Cardiol.* 50 598–605. 10.1016/j.yjmcc.2011.01.007 21262230

[B8] Heidenreich P. A. Albert N. M. Allen L. A. Bluemke D. A. Butler J. Fonarow G. C. (2013). Forecasting the impact of heart failure in the United States: a policy statement from the American Heart Association. *Circ. Heart Fail.* 6 606–619. 10.1161/HHF.0b013e318291329a 23616602 PMC3908895

[B9] Kannel W. B. Cobb J. (1992). Left ventricular hypertrophy and mortality–results from the Framingham study. *Cardiology* 81 291–298. 10.1159/000175819 1301257

[B10] Kolwicz S. C. Jr. Olson D. P. Marney L. C. Garcia-Menendez L. Synovec R. E. Tian R. (2012). Cardiac-specific deletion of acetyl CoA carboxylase 2 prevents metabolic remodeling during pressure-overload hypertrophy. *Circ. Res.* 111 728–738. 10.1161/CIRCRESAHA.112.268128 22730442 PMC3434870

[B11] Kundu B. K. Zhong M. Sen S. Davogustto G. Keller S. R. Taegtmeyer H. (2015). Remodeling of glucose metabolism precedes pressure overload-induced left ventricular hypertrophy: review of a hypothesis. *Cardiology* 130 211–220. 10.1159/000369782 25791172 PMC4394867

[B12] Kurdi M. Booz G. W. (2007). Can the protective actions of JAK-STAT in the heart be exploited therapeutically? Parsing the regulation of interleukin-6-type cytokine signaling. *J. Cardiovasc. Pharmacol.* 50 126–141. 10.1097/FJC.0b013e318068dd49 17703129

[B13] Lai L. Leone T. C. Keller M. P. Martin O. J. Broman A. T. Nigro J. (2014). Energy metabolic reprogramming in the hypertrophied and early stage failing heart: a multisystems approach. *Circ. Heart Fail.* 7 1022–1031. 10.1161/CIRCHEARTFAILURE.114.001469 25236884 PMC4241130

[B14] Mori J. Alrob O. A. Wagg C. S. Harris R. A. Lopaschuk G. D. Oudit G. Y. (2013). ANG II causes insulin resistance and induces cardiac metabolic switch and inefficiency: a critical role of PDK4. *Am. J. Physiol. Heart Circ. Physiol.* 304 H1103–H1113. 10.1152/ajpheart.00636.2012 23396452

[B15] Roger V. L. Go A. S. Lloyd-Jones D. M. Benjamin E. J. Berry J. D. Borden W. B. (2012). Heart disease and stroke statistics–2012 update: a report from the American Heart Association. *Circulation* 125 e2–e220. 10.1161/CIR.0b013e31823ac046 22179539 PMC4440543

[B16] Takeda K. Kaisho T. Yoshida N. Takeda J. Kishimoto T. Akira S. (1998). Stat3 activation is responsible for IL-6-dependent T cell proliferation through preventing apoptosis: generation and characterization of T cell-specific Stat3-deficient mice. *J. Immunol.* 161 4652–4660. 9794394

[B17] Wegrzyn J. Potla R. Chwae Y. J. Sepuri N. B. Zhang Q. Koeck T. (2009). Function of mitochondrial Stat3 in cellular respiration. *Science* 323 793–797. 10.1126/science.1164551 19131594 PMC2758306

[B18] Wu S. P. Kao C. Y. Wang L. Creighton C. J. Yang J. Donti T. R. (2015). Increased COUP-TFII expression in adult hearts induces mitochondrial dysfunction resulting in heart failure. *Nat. Commun.* 6 8245 10.1038/ncomms9245 PMC4568566 26356605

[B19] Yamasaki N. Kitaoka H. Matsumura Y. Furuno T. Nishinaga M. Doi Y. (2003). Heart failure in the elderly. *Intern. Med.* 42 383–388. 10.2169/internalmedicine.42.383 12793706

[B20] Yan J. Young M. E. Cui L. Lopaschuk G. D. Liao R. Tian R. (2009). Increased glucose uptake and oxidation in mouse hearts prevent high fatty acid oxidation but cause cardiac dysfunction in diet-induced obesity. *Circulation* 119 2818–2828. 10.1161/CIRCULATIONAHA.108.832915 19451348 PMC2765220

[B21] Zhao L. Cheng G. Jin R. Afzal M. R. Samanta A. Xuan Y. T. (2016). Deletion of interleukin-6 attenuates pressure overload-induced left ventricular hypertrophy and dysfunction. *Circ. Res.* 118 1918–1929. 10.1161/CIRCRESAHA.116.308688 27126808 PMC4902783

[B22] Zouein F. A. Altara R. Chen Q. Lesnefsky E. J. Kurdi M. Booz G. W. (2015). Pivotal importance of STAT3 in protecting the heart from acute and chronic stress: new advancement and unresolved issues. *Front. Cardiovasc. Med.* 2:36 10.3389/fcvm.2015.00036 PMC4671345 26664907

[B23] Zouein F. A. Zgheib C. Hamza S. Fuseler J. W. Hall J. E. Soljancic A. (2013). Role of STAT3 in angiotensin II-induced hypertension and cardiac remodeling revealed by mice lacking STAT3 serine 727 phosphorylation. *Hypertens. Res.* 36 496–503. 10.1038/hr.2012.223 23364341 PMC3674130

