# Supplementary material for: Cardiac STAT3 Deficiency Impairs Contractility and Metabolic Homeostasis in Hypertension
Source: Front Pharmacol. 2016 Nov 16;7:436. doi: 10.3389/fphar.2016.00436 (PMC5110511; doi:10.3389/fphar.2016.00436)
Supplement: Supplementary file 1 [file Data_Sheet_1.pdf]

Supplementary Figure Legends

Supplementary Figure 1 – The αMyHC-Cre expressing mice on a C57BL/6J background were obtained from The Jackson Laboratory (B6.FVB-Tg (Myh6-cre) 2182Mds/J). The colony was maintained as wild type (WT) and hemizygous for the *Cre* transgene. (A) Gene expression for αMyHC-Cre was assessed using *ATG ACA GAC AGA TCC CTC CTA TCT CC* as forward primer and *CTC ATC ACT CGT TGC ATC ATC GAC* as reverse primer with a PCR product of ~300 bp. Mice of lanes 1 and 4 are hemizygous for the *Cre* transgene; mice of lanes 2 and 3 lack the transgene (WT). (B) The mouse *STAT3* gene sequence was identified by the following primers: 1) *CCT GAA GAC CAA GTT CAT CTC TGT GAC*, 2) *CAC ACA AGC CAT CAA ACT CTG GTC TCC*, and 3) *GAT TTG AGT CAG GGA TCC TTA TCT TCG.* The WT *STAT3* gene produces a PCR product of 250 bp; the floxed gene, 350 bp. Mice of lanes 1 and 2 are heterozygous for a loxP-flanked allele; mice of lanes 3 and 4 are homozygous for floxed *STAT3*. The mouse of lane 4 is a STAT3 KO.

Western blots were done on heart lysates of WT and STAT3 KO mice. Blots were probed with an antibody recognizing the C-terminus (C) or the N-terminus (D) of STAT3. Blots were co-probed for GAPDH as loading control using a different species antibody. With the C-terminus antibody (Cell Signaling #9139), STAT3 levels were reduced >75% in STAT3 KO hearts (residual is from non-cardiomyocytes). No difference was seen with the N-terminus antibody (Santa Cruz Biotechnology #sc-7179). The gradient gel used did not permit discrimination based on the small size difference between the 2 STAT3 proteins. Results are representative of 3 WT and 3 STAT3 KO hearts.

Supplementary Figure 2 – Evidence of preserved cardiac function in WT and Cre^+ ANG II treated mice. A) Ejection fraction (EF) and B) fractional shortening (FS) were assessed by echocardiography. Measurements were performed on day 0, 14, and 28 and presented as the relative change from day 0. ****$p \leq 0.0001$, $n \geq 11$.

Suppl. Fig. 1

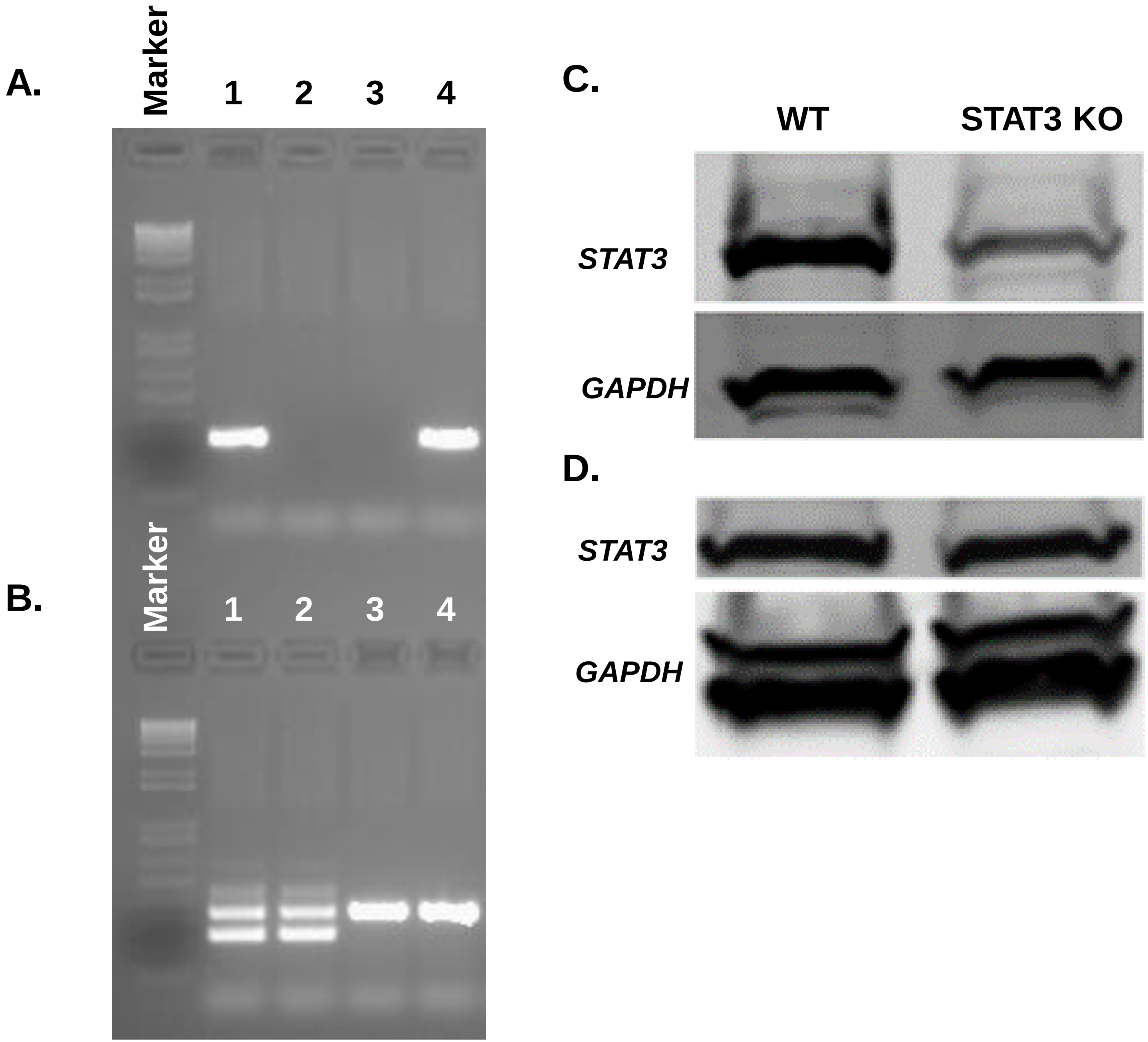
A.
Marker
1
2
3
4
B.
Marker
1
2
3
4
C.
WT
STAT3 KO
STAT3
GAPDH
D.
STAT3
GAPDH

Suppl. Fig. 2

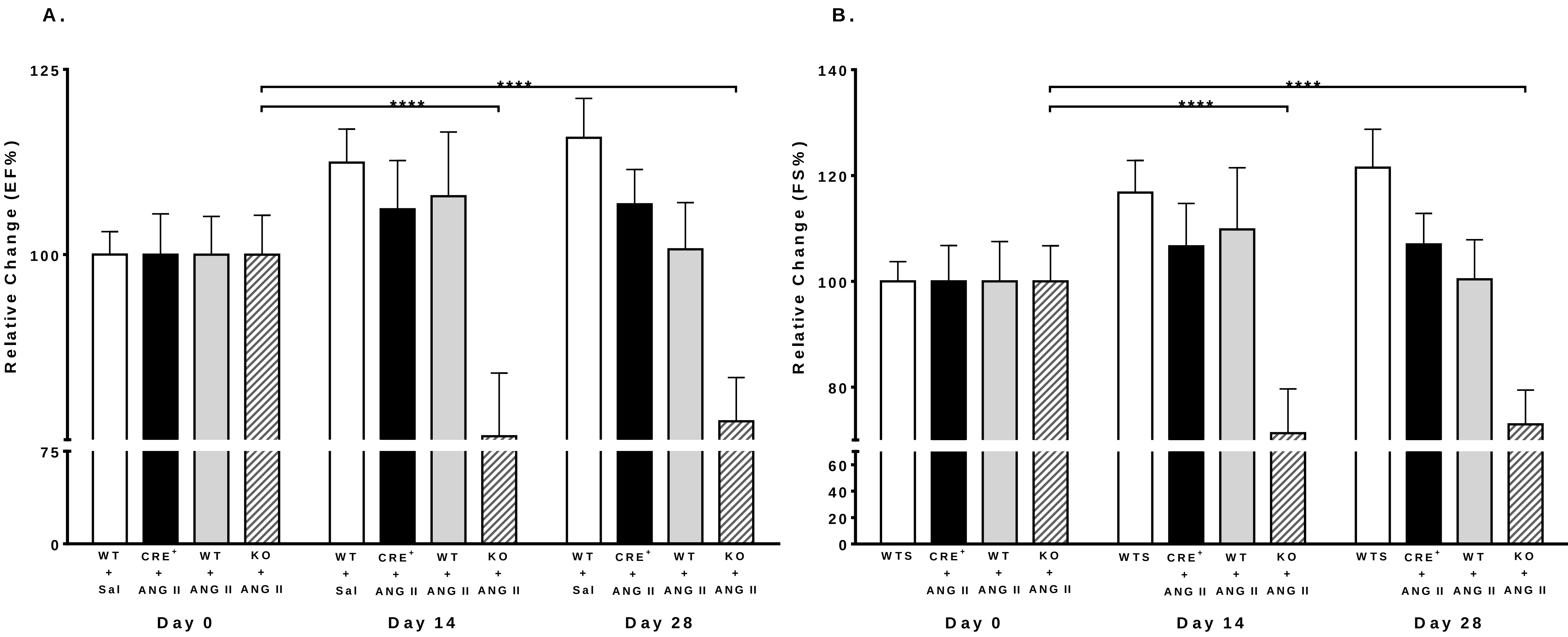
A.
Relative Change (EF%)
125
100
75
0
****
****
WT + Sal
CRE+ + ANG II
WT + ANG II
KO + ANG II
Day 0
Day 14
Day 28
B.
Relative Change (FS%)
140
120
100
80
60
40
20
0
WTS
Day 0
Day 14
Day 28
